# Developmental Exposure to Concentrated Ambient Particles and Preference for Immediate Reward in Mice

**DOI:** 10.1289/ehp.1205505

**Published:** 2012-10-11

**Authors:** Joshua L. Allen, Katherine Conrad, Günter Oberdörster, Carl J. Johnston, Brianna Sleezer, Deborah A. Cory-Slechta

**Affiliations:** 1 Department of Environmental Medicine, and; 2 Department of Pediatrics, University of Rochester School of Medicine, Rochester, New York, USA

**Keywords:** air pollution, ambient particles, attention deficit, behavior, delay of reward, fixed-ratio waiting-for-reward, impulsivity, particulate matter, ultrafine particles

## Abstract

Background: Recent epidemiological studies indicate negative associations between a diverse group of air pollutants and cognitive functioning in children and adults, and aspects of attention deficit in children. Neuroinflammation and oxidative stress are two putative biological mechanisms by which air pollutants may adversely affect the brain.

Objectives: We sought to determine whether exposure to concentrated ambient particulate matter (CAPS) during the first 2 weeks of life, alone or again in adulthood, could alter responding for delayed reward, a critical component of human decision making. Greater preference for immediate reward has been implicated as a component of several psychiatric disorders, addiction, obesity, and attention deficit.

Methods: C57BL/6J mice were exposed to ultrafine particles (< 100 nm in aerodynamic diameter; CAPS) using the Harvard University Concentrated Ambient Particle System (HUCAPS) or filtered air in the postnatal period (days 4–7 and 10–13) with and without adult exposure over days 56–60. In adulthood, delay behavior was assessed using a fixed-ratio waiting-for-reward (FR wait) paradigm in which 25 responses (FR25) were required to initiate the waiting-for-reward component during which mice obtained “free” sucrose pellets with the stipulation that these “free” pellets were delivered at increasing delay intervals.

Results: Coupled with increased FR response rates, mice exposed to postnatal CAPS displayed increased FR resets that reinstated short delays, indicating a preference for shorter delays, despite the added response cost of the FR25. No associated changes in locomotor activity were observed.

Conclusions: Postnatal CAPS exposure produces an enhanced bias towards immediate rewards, a risk factor for several central nervous system (CNS) disorders. This enhancement does not appear to be the result of hyperactivity. The findings underscore the need for further evaluation of air pollution effects on the CNS and its potential contribution to CNS diseases and disorders.

Ultrafine particles (UFP; < 100 nm in aerodynamic diameter) from multiple sources—ubiquitous in ambient urban and indoor air (Oberdörster et al. 2004)—are considered the more toxic component of ambient air pollution (Oberdörster 2000). UFP have been associated with adverse effects on the human cardiopulmonary system and with increased levels of circulating inflammatory markers (Delfino et al. 2005; Maier et al. 2008; Weichenthal et al. 2007). Although early development may represent a period of increased vulnerability of the lung to air pollutants (Delfino et al. 2005; Fanucchi et al. 2006; Frischer et al. 1999; Kulkarni et al. 2006; Mauad et al. 2008; Weichenthal et al. 2007), exposures to air pollutants are cumulative and occur across the life span.

Increasing evidence also suggests that air pollutants may adversely affect the central nervous system (CNS). Experimental data suggest UFP can enter the CNS directly (Oberdörster et al. 2002, 2004), producing inflammatory and oxidative responses similar to those seen in pulmonary tissue (Alessandrini et al. 2009; Delfino et al. 2009; Fujii et al. 2001; Ishii et al. 2004; Nemmar et al. 2009; van Eeden et al. 2001). For example, rodents subacutely exposed to diesel engine exhaust showed increased levels of proinflammatory and prooxidant markers such as tumor necrosis factor-α (TNF-α), interleukin-1α, Jun oncogene (AP-1), and nuclear factor kappa B (NFκB) in the striatum (Gerlofs-Nijland et al. 2010). Similar proinflammatory/oxidant effects of UFP were seen in apolipoprotein E knockout (ApoE^–/–^) mice (Campbell et al. 2009; Kleinman et al. 2008). Microglia, the resident macrophages in the brain, were shown *in vitro* to be primed in response to diesel exhaust exposure when later challenged with lipopolysaccharide (LPS) (Levesque et al. 2011). Rodents prenatally exposed to ozone exhibit disruptions in cerebellar monoaminergic neurotransmitters (Gonzalez-Pina et al. 2008), signs of cerebellar necrosis (Rivas-Manzano and Paz 1999), and loss of substantia nigra dopamine neurons (Santiago-López et al. 2010). Consistent with what is observed in the lung, the postnatal period of development may also represent a timeframe of increased susceptibility of the CNS to air pollutants. Notably, direct translocation of UFP into the brain would not be necessary to elicit neurotoxicity because peripheral inflammation itself can adversely affect the CNS (Banks et al. 2002; Delfino et al. 2009; Hagberg and Mallard 2005; Konsman et al. 2002; Perry 2004).

Behavioral functions also appear to be susceptible to air pollutants. Ambient black carbon, a marker of traffic-generated particulate matter, was associated with reduced neurocognitive functioning (verbal, nonverbal, and memory constructs) in urban school-age children (Suglia et al. 2008). Long-term black carbon exposure from ambient traffic pollution was associated with neurocognitive decline in men 71 ± 7 years of age (Power et al. 2011). Results from a recent cross-sectional study report a positive correlation between diagnosed attention deficit/hyperactivity disorder and ambient particulate matter level [odds ratio (OR) = 2.07; 95% CI: 1.08, 3.99] in school-age children (9–17 years of age) living in either urban or rural India after controlling for potential confounders (Siddique et al. 2011). Studies examining impacts of polycyclic aromatic hydrocarbon exposures report increases in anxiety and depression as well as attention problems (Perera et al. 2012) and reductions in IQ (Perera et al. 2009). Male mice exposed to air pollution for 10 months exhibited impaired cognition in a Barnes maze and increased floating in a Porsolt swim test, which is considered a model of behavioral despair (Fonken et al. 2011). Deficits in short-term memory have also been reported in male rats exposed to ambient air pollution (Zanchi et al. 2010).

To further understand the behavioral processes influenced by air pollution, particularly when it occurs early in development, in the present study we examined the influence of concentrated ambient particles (CAPS) exposure on the ability to sustain delays for free reward deliveries. Both humans and animal subjects typically prefer smaller immediate rewards over larger delayed rewards when given a choice (Broos et al. 2012b; Evenden and Ryan 1996; Peters and Buchel 2011). Delay discounting is a phenomenon in which rewards are increasingly devalued, that is, become less rewarding, as a function of the delay to their delivery (Peters and Buchel 2011). The discounting of delayed rewards is considered critical to human decision making, self-regulation, delay of gratification, and impulse control (Peters and Buchel 2011). Greater preference for immediate reward has been implicated in addictive behaviors including gambling, smoking, drug and alcohol abuse, obesity, conduct disorders, and attention deficit disorder (Bickel et al. 2012; Broos et al. 2012a). Mediation of such behavior has been tied to dopamine and glutamate neurochemical systems in the frontostriatal circuits (Peters and Buchel 2011), regions which have also been shown to be affected in various studies of air pollutants (Gerlofs-Nijland et al. 2010; Pereyra-Muñoz et al. 2006).

Given the epidemiological evidence suggesting that ambient air pollution is associated with cognitive dysfunctions and other behavioral impairments, as well as with adverse impacts in regions and neurotransmitters within the frontostriatal CNS circuitry, we sought to determine if CAPS exposure during early postnatal life in mice would alter responding to delayed reward assessed using a fixed-ratio waiting-for-reward (FR wait) paradigm previously shown to be sensitive to other toxicants (Bowen and McDonald 2009; Brockel and Cory-Slechta 1998, 1999). In addition, we examined whether any observed effects on the FR wait schedule might be enhanced by subsequent additional adult exposures or influenced by changes in activity levels.

## Materials and Methods

*Animals*. Young adult male and female C57BL/6J mice obtained from Jackson Laboratories (Bar Harbor, ME) were bred using a monogamous breeding scheme. Male and female mice were paired for 3 days, after which males were removed from the home cage. Pregnant dams (*n* = 63) remained singly housed with unculled litters throughout weaning. Litter size (mean ± SD) was 6.92 ± 1.9 and 54 ± 3% male. Pups were randomly assigned to treatment groups. To preclude litter-specific effects, a single pup per litter was used and litter size was counterbalanced when assigning litters to a treatment group. Other pups generated from the breeding cohort were allocated for other experimental purposes. Upon weaning at postnatal day (PND) 25, male progeny were group housed (2–3/cage) under a 12-hr reversed light/dark cycle and temperature maintained at 72°F. Weanling mice (*n* = 7–8/treatment group) were removed from the dams and individually exposed to CAPS or HEPA-filtered (99.99% effective) room air over PNDs 4–7 and 10–13 for 4 hr/day between 0700–1300 hours. The hours corresponded to high levels of vehicular traffic near the ambient air intake valve. These exposures were carried out in compartmentalized whole-body inhalation exposure chambers using the Harvard University Concentrated Ambient Particle System (HUCAPS) fitted with a size-selective inlet containing a high-volume (5,000 L/min) UFP concentrator to concentrate ambient particles. Filtered air and CAPS-treated mice experienced similar experimental manipulations. Particle counts were obtained using a condensation particle counter (model 3022A; TSI, Shoreview, MN) and mass concentration was calculated using idealized particle density (1.5 g/cm^3^). Ultrafine CAPS concentrations in the exposure chamber were approximately 10 times that of ambient air. Controls were exposed to filtered air in an adjacent chamber. Both CAPS and control mouse chambers were maintained at 77–79°F and 35–40% relative humidity. At PND56, mice received a secondary challenge with CAPS or filtered air for an additional 4 days to assess cumulative toxicity, generating four treatment groups: postnatal air with (*n* = 8) and without (*n* = 8) adult CAPS-exposure (designated Air/Air and Air/CAPS, respectively) and postnatal CAPS with (*n* = 7) and without (*n* = 7) adult CAPS exposure (designated CAPS/CAPS and CAPS/Air, respectively). Twenty-four hours after the final exposure, mice were moved into the behavioral suite and allowed to acclimate for 1 week prior to commencing behavioral testing on PND71. At this time, *ad libitum* weights were obtained and they averaged 30.2 g. All mice used in this study were treated humanely and with regard for alleviation of suffering.

*Operant behavior apparatus and procedure.* Behavioral testing was conducted in operant chambers (Med Associates, St. Albans, VT) housed in sound-attenuating cabinets equipped with white noise and fans for ventilation. Three levers were located horizontally across the back wall of the chamber, with a liquid dipper and dual pellet dispenser for reinforcer delivery on the front (opposite) wall. Mice were trained to press the left lever via an overnight autoshaping program in which they received liquid reinforcer (0.2% saccharine solution). Subsequently, a fixed ratio (FR) schedule was imposed, requiring a fixed number of responses for each reward delivery, with the response requirement increased across training sessions until 25 responses (FR25) were required to earn each reward. Sessions lasted 30 min or until 30 reinforcers had been earned, whichever occurred first. Liquid reward was used for autoshaping and FR3, after which a transition to 20-mg sucrose pellets (BioServ, Frenchtown, NJ) as a reinforcer was made because of low rates of responding for saccharin. When using liquid reinforcement, animals were maintained on a water schedule in which they received free access to water for 4 hr after completion of a behavioral session. When using sucrose pellet reinforcers, adult progeny were maintained at 85% *ad libitum* weight (23–29 g; mean 25.7 g) to facilitate behavioral testing.

After 20 sessions under the FR25 schedule—​at which time all mice had reliably earned all 30 reinforcers/session in the last three sessions—a waiting-for-reward component was added to the schedule. The wait component allowed the mice to obtain “free” sucrose pellets after completing the FR25, with the stipulation that these “free” pellets were delivered at increasing delay intervals, with the interval length varying across sessions (5, 7.5, 10, 12.5, or 15 sec) in a pseudo-random pattern. For instance, in a session utilizing a 7.5-sec latency (wait time), the first free reinforcer was delivered at 7.5 sec, then 15 sec, then 22.5 sec … and so on. Initial values were set to be shorter than the average post-reinforcement pause (PRP) time (time to first response in each ratio) to ensure mice made contact with the wait component contingencies. There was no upper limit for the waiting component except session termination. Emission of a lever press response during the wait component, however, reset the FR25 requirement for initiating the next wait component. Behavioral sessions were conducted during the dark cycle, 4 days/week, and were 20 min in duration.

*Locomotor behavior apparatus and procedure.* To evaluate hyperactivity, spontaneous locomotor activity was measured in photobeam chambers equipped with a transparent acrylic arena) with a 48-channel infrared source, detector, and controller (Med Associates). Locomotor activity was quantified in two 45-min sessions occurring once per day on 2 consecutive days after FR25 training (at approximately 6 months of age), but prior to the addition of the wait component to the FR wait. Ambulatory counts are defined as number of episodes in which a mouse broke three successive horizontal photobeams, whereas vertical counts are number of *z*-axis photobeam (located 5.25 cm above the arena floor) breaks.

*Data and statistical analysis.* FR response rate was defined as the total number of FR responses divided by the total time spent in the FR component, and run rate as total number of FR responses divided by the time spent in the FR component minus PRP time. Mean wait time for a “free” pellet was equal to the average wait time across wait components of a session, and longest wait time to the mean of the longest wait times for a “free” pellet across wait components in a session. Interresponse time (IRT) was defined as the median time lapsed between lever responses. Reinforcement density was defined as total number of reinforcers/session time. FR resets was equal to the total number of times the wait component was ended by a response, resetting the FR requirement. Responses per reinforcer (both free and FR reinforcers) and a ratio of mean wait time divided by the time to complete an FR25 were used as measures of behavioral “efficiency.”

After three adaptation sessions to the FR wait schedule, the final nine behavioral sessions were analyzed. These included one session each for 5.0- and 12.5-sec, two sessions using 7.5- and 10.0-sec, and three sessions using 15.0-sec wait intervals. Cumulative records were generated using SoftCR Pro software (Med Associates), in which session time is displayed along the *x*-axis of the cumulative record and lever responses cumulated on the *y*-axis, returning to baseline after each 30 responses.

Repeated measures analysis of variance (RMANOVA) with postnatal and adult treatment as the between group factors and session (locomotor activity, FR baseline) or wait interval (FR wait; mean values used for 7.5-, 10-, and 15.0-sec wait intervals) as the within factor were used to analyze data. Results were followed as appropriate depending on main effects or interactions, by Fisher’s protected least-squares differences (PLSDs) test. Statistical analyses were conducted using Statview version 5.0 (SAS Institute Inc., Cary, NC), with *p* < 0.05 considered statistically significant and one *p* = 0.056 trend indicated. The statistical outcomes are summarized in Supplemental Material, Table S1 (http://dx.doi.org/10.1289/ehp.1205505).

## Results

**Figure 1:**
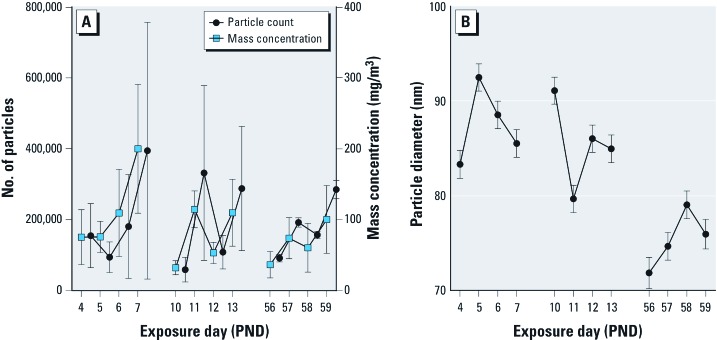
Exposure conditions for animals exposed to CAPS during the early postnatal, young adult, or both exposure periods. Changes in mean particle count (*A*; left *y*-axis) closely paralleled changes in mean particle mass concentration (*A*; right, *y*-axis). Particles remained < 100 nm (ultrafine) during all exposures (*B*). Data are reported as group means ± SDs.

*Exposure conditions.* Mean particle count for 8 days of postnatal exposure was 2.02 × 10^5^ particles/m^3^, whereas mean daily particle mass concentration was 96.4 µg/m^3^ and mean daily particle count for 4 days of adult exposure was 1.82 × 10^5^ particles/m^3^ with a mean mass concentration of 67.9 µg/m^3^ [Figure 1A; see also Supplemental Material, Table S2 (http://dx.doi.org/10.1289/ehp.1205505)]. Particles remained ultrafine (< 100 nm) in size across exposure days for both postnatal and adult exposures (Figure 1B). Ultrafine CAPS concentrations in the exposure chambers were approximately 10 times that of ambient air from which the particles were concentrated. Ambient levels of UFP were similar to those reported for Erfurt (Germany) and Atlanta (Georgia), which ranged from approximately 10,000–20,000 particles/cm^3^ with 24-hr maxima of 50,000 particles/cm^3^ (Wichmann and Peters 2010; Woo et al. 2010).

Crude estimates of the inhaled doses of CAPS [see Supplemental Material, Table S2 (http://dx.doi.org/10.1289/ehp.1205505)] suggest that mice received a total of approximately 308.5 and 233.6 μg CAPS/kg body weight in the postnatal and adult exposure periods, respectively. Animals in the CAPS/CAPS group received an estimated total dose of 542.1-μg/kg body weight over both exposure periods.

*Autoshaping and baseline FR performance.* All mice learned to lever press within five sessions. No systematic treatment-related differences in the number of sessions required to autoshape were observed. Group mean FR overall response rates increased across baseline sessions from approximately 20–50 to 50–75 responses/min [see Supplemental Material, Figure S1 (http://dx.doi.org/10.1289/ehp.1205505); *F*(18, 468) = 24.35, *p* < 0.0001], but did not differ by treatment group. Mean FR run rates also increased across sessions from approximately 80–200 to 150–200 responses/min [*F*(18, 468) = 17.28, *p* < 0.0001]. Run rates were characterized by an interaction of postnatal × adult treatment × sessions [*F*(18, 468) = 2.92, *p* < 0.0001], but group post hoc comparisons did not reveal any significant systematic treatment-related differences. PRP time included a significant interaction between adult treatment by session [*F*(18, 468) = 1.84, *p =* 0.02], which reflected shorter PRP times in the CAPS/CAPS relative to Air/Air and Air/CAPS groups in post hoc tests. FR completion time decreased across sessions from approximately 30–45 to 20–35 sec [*F*(18, 468) = 17.13, *p* < 0.0001], but was not influenced by treatment conditions. IRTs decreased over sessions, from approximately 0.4–0.7 to 0.3–0.45 sec [*F*(18, 468) = 7.771, *p* < 0.0001], but no significant treatment-related differences were observed. No treatment-related differences were found in total numbers of reinforcers earned, total session time, or reinforcement density (data not shown).

**Figure 2:**
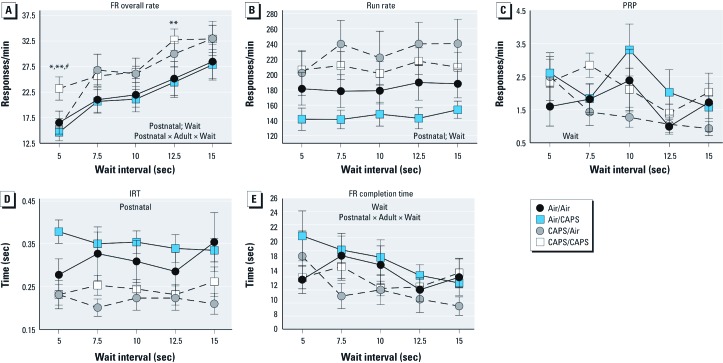
FR overall response rates (*A*), run rates (*B*), PRP (*C*), IRT (*D*), and FR completion time (*E*) in relation to wait interval during the FR waiting-for-reward schedule for mice exposed to CAPS or filtered air in postnatal, adult, or both exposure periods as indicated. Data are reported as group means ± SEs, of one session each for 5.0 and 12.5 sec, and the mean of two sessions of 7.5 and 10.0 sec and three sessions at 15.0 sec; *n* = 7–8/treatment group. *p < 0.05 compared with Air/Air animals; **p < 0.05 compared with Air/CAPS-treated animals; *p* < 0.05 compared with CAPS/Air-treated animals.

*FR waiting-for-reward: FR component performance*. As the wait interval increased, FR overall rate and run rate significantly increased, while PRP and FR completion time decreased [Figure 2; see also Supplemental Material, Table S1 (http://dx.doi.org/10.1289/ehp.1205505)]. Postnatal CAPS treatment significantly increased FR component response rates by 18–43% (postnatal: *F*(1, 26) = 4.64, *p* = 0.04; postnatal × adult treatment × wait [*F*(4, 104) = 2.619, *p* = 0.04; Figure 2A, dotted lines]. Increases in CAPS/CAPS-treated mice compared to all other groups were found at the 5-sec wait interval, and as compared to the CAPS/Air group at the 12.5-sec wait (*p* < 0.05). A similar postnatal CAPS-induced increase in run rates (11–33%) was observed [Figure 2B; *F*(1, 104) = 7.12, *p* = 0.013] that occurred across wait intervals. Correspondingly, postnatal CAPS reduced IRTs by approximately 27% across intervals [Figure 2D; *F*(1, 26) = 10.05, *p* = 0.004]. FR completion time was characterized by a significant interaction of postnatal × adult treatment × wait interval [Figure 2E; *F*(4, 104) = 3.6, *p* = 0.009], which reflected faster FR completion times, particularly for the CAPS/Air group. No treatment-related differences in PRP times were observed (Figure 2C).

**Figure 3:**
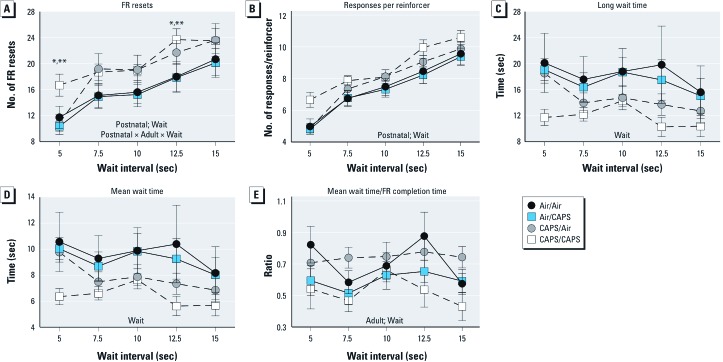
FR resets (*A*), responses per reinforcer (*B*), long wait time (*C*), mean wait time (*D*), and mean wait time/FR completion time (*E*) for mice exposed to CAPS or filtered air in postnatal, adult, or both exposure periods acquired during FR waiting-for-reward sessions, as indicated. Data are reported as group means ± SEs, of one session each for 5.0 and 12.5 sec, and the mean of two sessions of 7.5 and 10.0 sec and three sessions at 15.0 sec; *n* = 7–8/treatment group. *p < 0.05 compared with Air/Air animals; **p < 0.05 compared with Air/CAPS-treated animals.

*FR waiting-for-reward: waiting behavior.* FR resets, and responses/reinforcer significantly increased with wait interval, whereas mean wait, and long wait times as well as mean wait time/FR completion time, decreased [Figure 3; see also Supplemental Material, Table S1 (http://dx.doi.org/10.1289/ehp.1205505)]. CAPS/CAPS treatment significantly increased the number of FR resets during both the 5-sec (42%) and 12.5-sec (30%) wait interval sessions compared to Air/Air and Air/CAPS groups, which had similar but nonsignificant effects at 7.5 and 10 sec {main effect of postnatal CAPS [Figure 3A; *F*(1, 26) = 4.56, *p* = 0.042]; interaction of postnatal × adult treatment × wait interval [*F*(4, 104) = 3.06, *p* = 0.02)}. Analyses of responses/reinforcer showed a nearly significant effect of postnatal treatment [Figure 3B; *F*(1, 104) = 3.99, *p =* 0.056], likely also due to CAPS/CAPS increases at the 5- and 12.5-sec wait interval sessions. No statistically significant treatment-related differences in long wait time (Figure 3C) or in mean wait time (Figure 3D) were observed; however, postnatal CAPS-treated mice had lower, albeit, not statistically significantly, long wait times and mean wait times. A main effect of adult treatment on the mean wait time/FR completion time ratio was observed [Figure 3E; *F*(1, 26) = 5.7, *p* = 0.03], reflecting the generally overall lower mean wait time/FR completion time ratios, collapsed across wait intervals for the CAPS/CAPS and Air/CAPS groups compared to the Air/Air and CAPS/Air groups.

**Figure 4:**
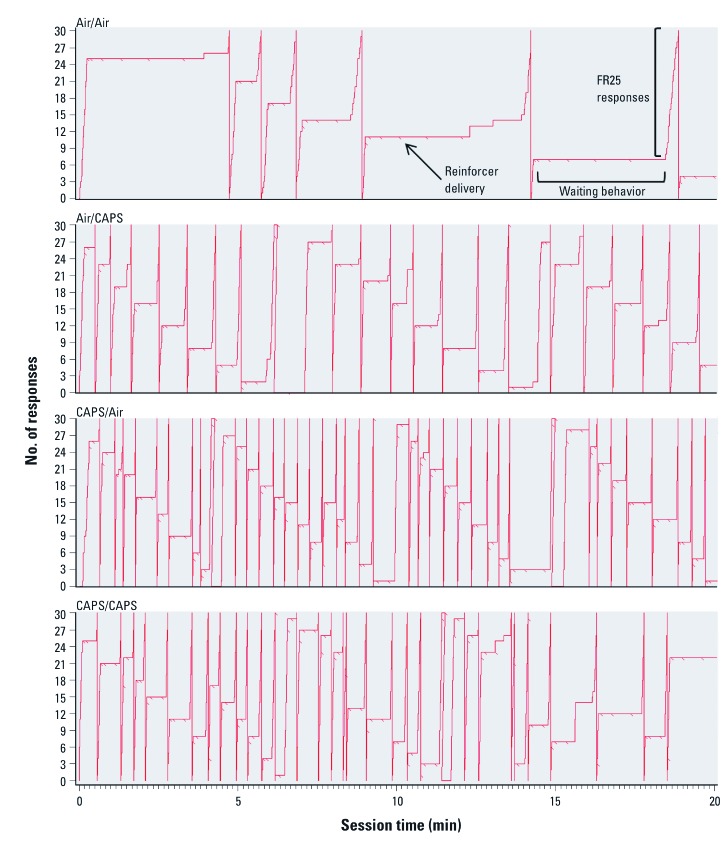
Cumulative records of responding for animals exposed to CAPS or filtered air in postnatal, adult, or both exposure periods while last 15-sec “wait” latency was in effect. The response counter automatically reset to baseline (0 responses) after each 30 responses occurs. As labeled, pips (backslashes) indicate reinforcer delivery, horizontal lines indicate periods of waiting, and vertical lines show FR responding (*n* = 7–8/treatment group).

Figure 4 depicts representative cumulative records of one mouse from each treatment group from the last session in which the 15-sec latency between “free” reinforcer deliveries was in effect. The records demonstrate both the high FR response output, depicted as a greater number of cumulative record pen resets to baseline, and the reduced waiting behavior (shorter and fewer horizontal lines) in CAPS-treated animals compared to air-treated animals.

*Locomotor activity.* Both horizontal [*F*(1, 26) = 84.18, *p* < 0.0001] and vertical activity [*F*(1, 26) = 26.3, *p* < 0.0001] decreased across sessions [see Supplemental Material, Figure S2 (http://dx.doi.org/10.1289/ehp.1205505)], but no treatment-related effects were observed.

## Discussion

This study demonstrates that postnatal CAPS treatment alters behaviors related to mediating delay of reward, here assessed on an FR waiting-for-reward schedule of reinforcement. As seen in the cumulative records in Figure 4, CAPS treatment, particularly when imposed in the postnatal period, increased FR response rates and resets of the FR component. Mean and long wait times were suggestively, but not significantly, lower after postnatal CAPS, consistent with the increased FR resets. The effect of adult exposures on mean wait time/FR completion time in part reflected the lower ratios of CAPS/CAPS-treated mice at long wait intervals; a decreased ratio indicated early resetting. The increase in FR resets demonstrated with postnatal CAPS suggests a preference for shorter rather than long delays, even if it requires the added response cost of the completion of an additional FR of 25 responses. Similar alterations in waiting behavior were seen in lead-treated rats (Brockel and Cory-Slechta 1998, 1999) and toluene-treated mice (Bowen and McDonald 2009).

This pattern of behavior has many similarities to delay discounting, in which the value of rewards declines as a function of the delay in their receipt, a phenomenon shown widely in both humans and experimental models. Here response cost (effort) was varied, with higher response cost associated with more immediate free reward and lower response cost with delayed free reward; in studies using a similar approach, low doses of amphetamine increased the tendency for rats to work harder in a task providing a choice between 1 lever press producing 2 pellets vs. 2–20 presses for 4 pellets, consistent with the importance of dopaminergic mediation (Floresco et al. 2008). The discounting of delayed rewards, sometimes considered analogous to impulsivity (but see also Broos et al. 2012b) and delay of gratification, is considered critical to human decision making, and has been related to addiction-related behaviors and other psychiatric conditions (Bickel et al. 2012) and has been shown to be a relatively stable trait in individuals across life (Casey et al. 2011). Impaired delay discounting has also been related to obesity (Bickel et al. 2012), an outcome recently related to prenatal exposures to the air pollutant diesel exhaust (Bolton et al. 2012).

Of course it is critical to consider other possible explanations for these effects. Increased FR resets and response rates could reflect the increase in earned reinforcers in postnatal CAPS-exposed mice compared to postnatal air-exposed mice (data not shown), which could have increased reinforcement density. This explanation seems unlikely, however, for several reasons. First, the increase in reinforcers averaged approximately 10%, and in absolute numbers constituted approximately 7 additional reinforcers per session. When considered across 20 min, this is equivalent to only 0.35 reinforcers/min. Additionally, the increase in reinforcers occurred at the cost of increased behavioral output, as evidenced by the nearly significant increase in number of responses required per reinforcer with postnatal CAPS treatment (Figure 3), consistent with an inefficient response pattern and over-expenditure of energy. Further, neither FR overall rates, run rates, FR completion time, nor responses per reinforcer systematically differed by treatment group during FR baseline sessions, with postnatal CAPS exposure effects emerging only after the wait component was added to the schedule.

The possibility that these effects simply reflected hyperactivity was assessed by measuring locomotor behavior. However, when spontaneous locomotor activity was quantified over a period of 2 days, no hyperactivity was observed, at least as measured in an open field. Whether it occurs under other conditions remains an open question. Collectively, these observations suggest that the higher FR resets and FR rates were the result of waiting for reward, rather than higher FR rates as the source of increased FR resets.

Thus considered relative to these alternatives, the findings appear to be more consistent with the interpretation that CAPS-treated animals preferred more immediate reinforcement, even though associated with a greater response cost, relative to free but delayed rewards. Increasing delays to reinforcer delivery may have been aversive to CAPS-treated animals and termination of this aversive stimulus was achieved by reinitiation of the FR component, which also decreased the delay to reward. Assessment of the effects of postnatal CAPS on other behavioral paradigms associated with ability to manage delay of reward and impulsivity (i.e., self-control paradigms, differential reinforcement of low rate schedule) will be useful to determine the generality of these effects and further understand their behavioral mechanisms.

One additional consideration of the differential responses to the FR wait schedule produced by postnatal CAPS exposure is that it also reflects cognitive dysfunction, that is, an inability to associate behavior with its consequences. Adverse CNS effects of ambient air pollutants on cognition have been reported. Negative associations of cognitive function with exposure to black carbon in a cohort of aged men (Power et al. 2011), and of fine (≤ 2.5 µm) and coarse particulate matter (2.5–10 µm) exposure with more rapid cognitive decline in aged women (Weuve et al. 2012) have been reported. A negative association of black carbon levels in the air with measures of cognition was reported in a cohort of Boston children (Suglia et al. 2008). In mice, exposure to fine ambient particulate matter caused depressive-like behavior, impaired spatial learning and memory, and produced hippocampal proinflammatory cytokine expression (Fonken et al. 2011).

Oxidative damage and neuroinflammation have been posited as underlying mechanisms by which air pollutants adversely affect the brain (Block and Calderón-Garcidueñas 2009). Interestingly, air pollutants have been shown to elicit a neuroinflammatory response in brain regions known to be involved in executive/cognitive functions (Elder et al. 2006; Fonken et al. 2011). Oxidative stress in the striata of ozone-treated rats (Pereyra-Muñoz et al. 2006) and neuroinflammation of the striata in rats exposed to diesel engine exhaust (Gerlofs-Nijland et al. 2010) have been reported. Histopathological, biochemical, and neurochemical changes in response to CAPS-exposure in the tissues generated from mice in this study and their relationship to the behavioral outcomes as well as composition of CAPS are currently under examination.

Because dose, duration of exposure, and time postexposure to behavioral testing differed between the postnatal and adult exposures, the ability to assess age-dependent and/or cumulative toxicity is limited. Therefore, whether postnatal CAPS-associated behavioral toxicity is further enhanced by subsequent adult exposure, or as compared with adult-only exposure, cannot be fully ascertained from these findings and warrants additional study.

However, two observations are notable. First, with respect to the FR wait, increases in FR rate and in FR resets showed evidence of being more pronounced in the CAPS/CAPS than CAPS/Air group, suggesting some additional vulnerability of cumulative exposures. Further, it is notable that postnatal CAPS was associated with behavioral toxicity that was present several months following the end of exposures, suggesting that developmental CAPS exposures can have protracted effects, thereby underscoring the need for further evaluation of air pollution effects on the CNS and how such exposures may contribute to CNS diseases and disorders.

## Supplemental Material

(393 KB) PDF
