# Supplementary material for: Developmental Exposure to Concentrated Ambient Particles and Preference for Immediate Reward in Mice
Source: Environ Health Perspect. 2012 Oct 11;121(1):32–8. doi: 10.1289/ehp.1205505 (PMC3553438; doi:10.1289/ehp.1205505)
Supplement: (393 KB) PDF [file ehp.1205505.s001.pdf]

Supplemental Material

Developmental Exposure to Concentrated Ambient Particles and Preference for Immediate Reward in Mice

Joshua L. Allen,[1] Katherine Conrad,[1] Günter Oberdörster,[1] Carl J. Johnston,[2] Brianna Sleezer,[1] and Deborah A. Cory-Slechta[1]

[1]Department of Environmental Medicine, and [2]Department of Pediatrics, University of Rochester School of Medicine, Rochester, New York, USA

Table of Contents:

Page 2: Figure S1: Baseline FR25 data

Page 3: Figure S2: Spontaneous locomotor activity data

Page 4: Table S1: Summary of statistical main effects and interactions for baseline FR25 data and FR waiting-for-reward data

Page 5: Table S2: Estimate of inhaled dose

Correction

In the manuscript originally published online, an author was omitted. Carl J. Johnston and his affiliation have been added here.

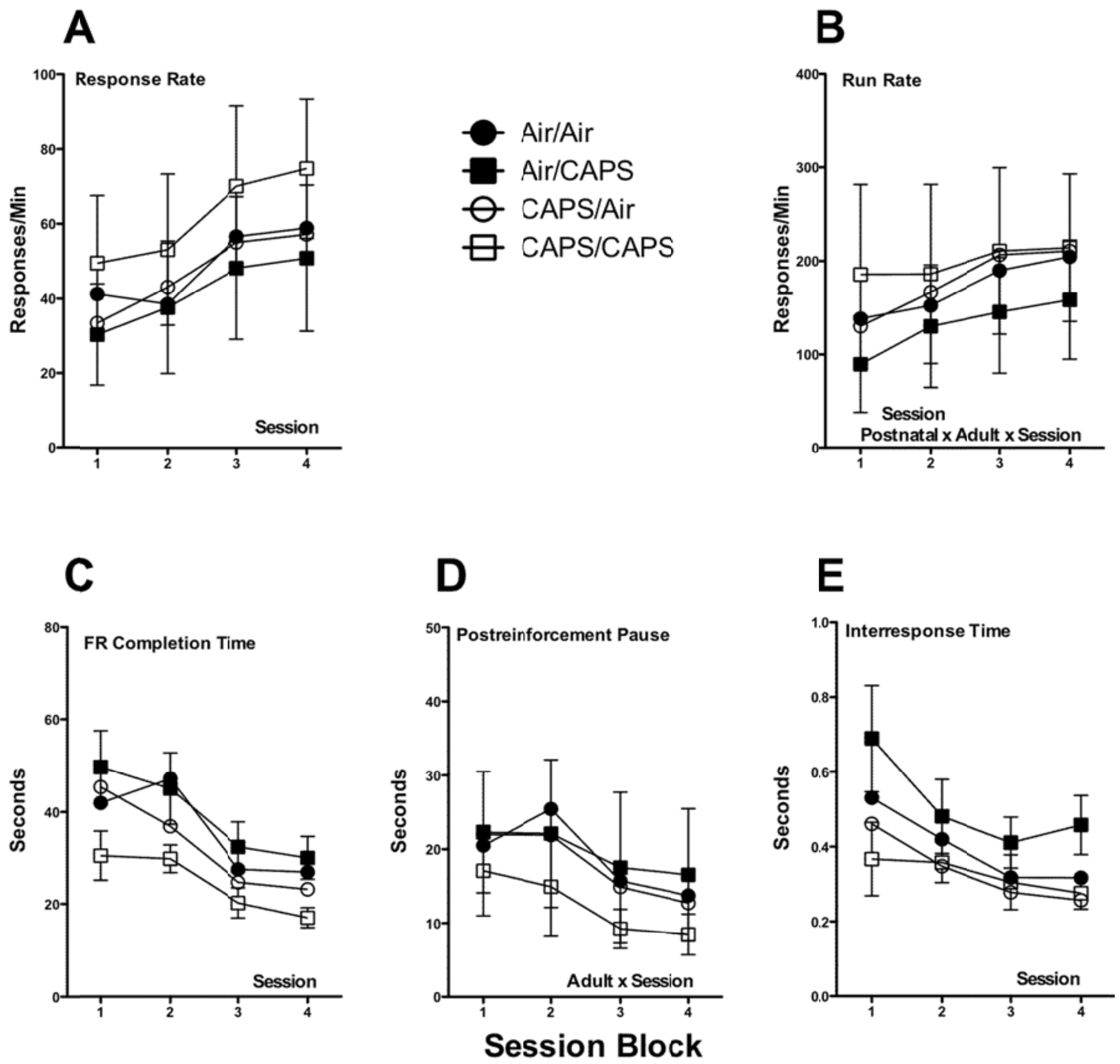

Figure S1. A. Response rates, **B.** Run rates, **C.** FR completion time, **D.** Post-reinforcement pause times (D), and **E.** Inter-response times (E) for mice exposed to CAPS in postnatal, adult, or both exposure period during FR25 baseline sessions. Five individual sessions were combined into a single data point. Reported as group mean ± SE, *n* = 7-8/treatment group. Labels above the x axis indicate outcome of statistical analyses.

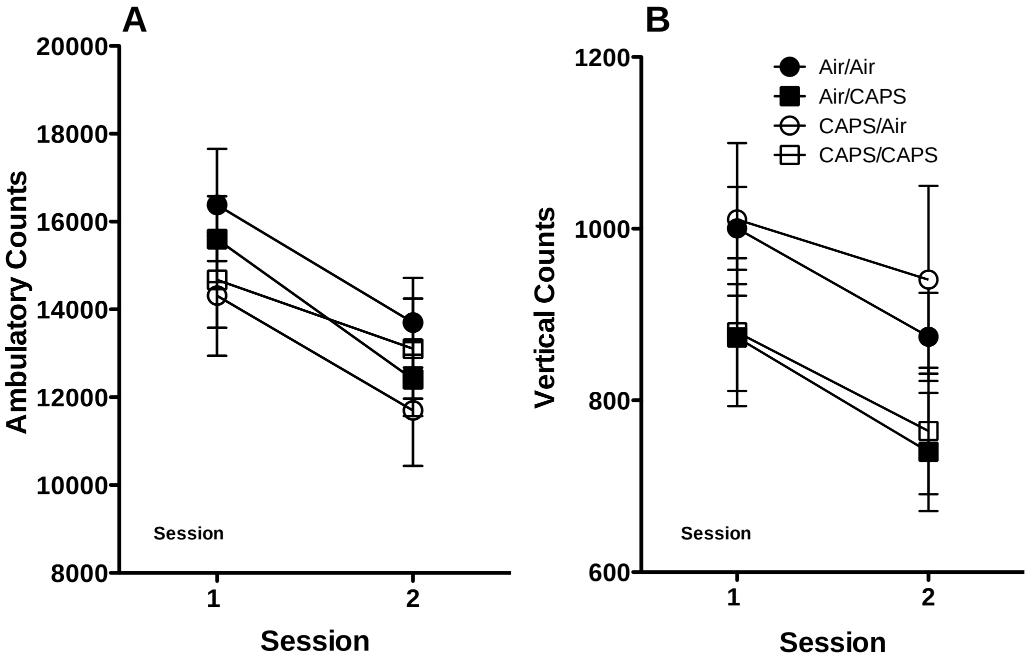

Figure S2. Horizontal and vertical spontaneous activity from 2 sessions from mice exposed to CAPS or filtered air in postnatal, adult, or both exposure periods. Data acquired for 2 days following cessation of final FR 25 training/baseline session. Reported as group mean ± SE, *n* = 7-8/treatment group. **A.** Ambulatory (horizontal movement) activity across sessions. **B.** Vertical (rearing) activity across sessions. Labels above the x axis indicate outcome of statistical analyses.

Table S1: Summary of two-factor repeated measures ANOVA results for FR and waiting behavior collected from FR baseline performance and FR waiting-for-reward sessions. $P<0.05$, unless otherwise indicated.

	FR Behavior				
	Overall Rate	***Run Rate***	***PRP***	***IRT***	***FR Comp***
FR Baseline	Session	Postnatal x Adult x Session; Session	Adult x Session	Session	Session
FR Waiting-For-Reward	Postnatal; Postnatal x Adult x Wait; Wait	Postnatal; Wait	Wait	Postnatal	Postnatal x Adult x Wait; Wait

	Waiting Behavior				
	FR Resets	***Resp/Reinf***	***Long Wait Time***	***Mean Wait Time***	***Mean Wait Time/FR Completion Time***
FR Waiting-For-Reward	Postnatal; Postnatal x Adult x Wait; Wait	Postnatal p=0.056; Wait	Wait	Wait	Adult; Wait

Table S2: Estimate of Inhaled Dose

Exposure Period	***Early Postnatal***	***Adult***
Average Mass Concentration (μg/m³)	96.4	67.9
Assumed Minute Ventilation (mL/min)	10[*]	86[+]
Duration of Exposure (min)	1920	960
Assumed Body Weight (g)	6[>]	24[<]
Estimated Total Inhaled Dose (ug)	1.9	5.6
Estimated Inhaled Dose (ug/kg body weight)	308.5	233.6

[*]Berner J, Shvarev Y, Lagercrantz H, Bilkei-Gorzo A, Hokfelt T, Wickstrom R. 2007. Altered respiratory patter and hypoxic response in transgenic newborn mice lacking the tachykinin-1 gene. J Appl Physiol 102: 552-559.

[+]http://phenome.jax.org/db/qp?rtn=views/measplot&brieflook=35101

[>] Middaugh LD, Randall CL, Favara JP. Prenatal ethanol exposure in C57 mice: Effects on pregnancy and offspring development. 1988. Neurotoxol Teratol 10(2): 175-180.

[<]http://jaxmice.jax.org/support/weight/000664.html
